# Skin Microbiome Patterns Associated with Basal Cell Carcinoma: A Case Series

**DOI:** 10.3390/microorganisms14040822

**Published:** 2026-04-03

**Authors:** Mavra Masood, David Ozog, Tengfei Ma, Marissa Ceresnie, Aunna Pourang, Christine C. Johnson, Xinyue Qiu, Albert Levin, Jesse Veenstra

**Affiliations:** 1 Department of Dermatology, Virginia Commonwealth University Health System, Richmond, VA 23298, USA; 2 Department of Dermatology, Henry Ford Health, Detroit, MI 48202, USA; 3 Department of Nutrition and Health Science, Ball State University, Muncie, IN 47306, USA; 4 Department of Dermatology, Lake Granbury Medical Center, Granbury, TX 76048, USA; 5 Department of Dermatology, Wayne State University, Detroit, MI 48202, USA; 6 Department of Public Health Sciences, Henry Ford Health, Detroit, MI 48202, USA

**Keywords:** basal cell carcinoma, microbiome, whole genome shotgun sequencing, *Cutibacterium acnes*

## Abstract

Basal cell carcinoma (BCC) is the most common malignancy worldwide, yet the role of the skin microbiome in BCC remains poorly defined. In this cross-sectional observational case series, we compared the cutaneous microbiome of BCC lesions with matched perilesional and control skin using whole-genome shotgun sequencing in an intra-patient, multi-site sampling design. BCC samples demonstrated reduced microbial richness and significant shifts in community composition compared with matched control skin. Specifically, BCC lesions exhibited significantly lower Chao1 diversity (β = −484.6, 95% CI: −772.1 to −197.2, *p* = 0.003). Differences in overall microbial composition were confirmed by PERMANOVA analysis based on Bray–Curtis and Jaccard distance metrics (R^2^ = 12.6% and 9.7%, respectively; both *p* = 0.01). At the species level, *Cutibacterium acnes* was significantly reduced in BCC samples compared with controls (β = −0.31, 95% CI: −0.45 to −0.16, *p* = 0.0004), corresponding to an approximately 27% lower geometric mean relative abundance. Functional profiling suggested shifts in microbial metabolic potential, with pathways related to redox balance and lipid-associated processes differentially represented in BCC samples relative to controls. Together, these findings demonstrate that BCC lesions are associated with localized alterations in microbial diversity, community composition, and inferred functional potential. These results support the presence of a tumor-associated microbiome signature in BCC; however, further studies in larger and more diverse cohorts are needed to determine whether these changes contribute to tumor development or reflect adaptation to the tumor microenvironment.

## 1. Introduction

The human skin microbiome is a complex and site-specific ecosystem composed of bacteria, fungi, and viruses that play essential roles in maintaining cutaneous homeostasis. These microorganisms contribute to barrier integrity, immune regulation, and metabolic processes through dynamic interactions with the host. The composition and function of the skin microbiome are shaped by both intrinsic factors, including host genetics, immune tone, and sebaceous activity, and extrinsic influences such as ultraviolet (UV) radiation, environmental exposures, and topical products [1,2]. Disruption of this equilibrium, or dysbiosis, has been implicated in a range of dermatologic conditions, including inflammatory dermatoses and cutaneous malignancies [1,3].

Functional studies further suggest that commensal-derived products, including extracellular vesicles, can modulate inflammation and barrier function, supporting a biologically plausible role for the microbiome in cutaneous disease processes [4]. Increasing evidence suggests that the skin microbiome may influence carcinogenesis through multiple mechanisms, including modulation of local immune responses, alteration of antimicrobial peptide signaling, and regulation of oxidative stress [3,5].

In keratinocyte carcinogenesis, studies in actinic keratosis and cutaneous squamous cell carcinoma (cSCC) have demonstrated microbial shifts characterized by reduced commensal diversity and overrepresentation of pro-inflammatory taxa such as *Staphylococcus aureus* [6,7]. These changes have been linked to enhanced expression of antimicrobial peptides and inflammatory mediators, supporting a role for microbiome-driven immune dysregulation in tumor-promoting microenvironments [7,8,9].

In parallel, commensal organisms such as *Cutibacterium acnes* have been implicated in maintaining cutaneous homeostasis through lipid metabolism, pH regulation, and modulation of inflammatory signaling pathways [10]. Notably, *C. acnes* produces the antioxidant protein RoxP, which contributes to protection against oxidative stress in the skin microenvironment [11,12].

Despite these advances, the role of the microbiome in basal cell carcinoma (BCC) remains poorly defined. BCC is the most common malignancy worldwide and is associated with substantial morbidity and healthcare burden [13,14]. It arises in the context of chronic UV exposure, aging, and local immune modulation [15]. While these risk factors are well established, the extent to which microbial communities contribute to or reflect the tumor microenvironment in BCC remains underexplored. It remains unclear whether microbial alterations in BCC represent passive changes within the tumor microenvironment or reflect biologically meaningful shifts with potential functional relevance.

In this cross-sectional observational study, we performed whole-genome shotgun sequencing to characterize the cutaneous microbiome across BCC lesions, matched perilesional skin, and anatomically comparable control sites within the same individuals. This intra-patient, multi-site sampling design enables assessment of tumor-associated microbial changes while minimizing inter-individual variability, thereby allowing more precise characterization of microbiome alterations localized to BCC lesions. In addition to taxonomic profiling, we evaluated microbial functional potential through metagenomic pathway analysis. We hypothesized that BCC lesions would exhibit localized shifts in microbial diversity, alterations in community composition, and distinct functional signatures compared to matched non-lesional skin.

## 2. Materials and Methods

### 2.1. Patient Samples

Study participants were identified through chart review based on histopathologically confirmed basal cell carcinoma (BCC; ICD-10: C44.X1) and a scheduled Mohs surgery appointment between 1 May 2022 and 1 May 2023 at Henry Ford Health, Novi, MI, USA. Eligible participants were aged 40 years or older, consistent with the increased incidence of BCC and cumulative ultraviolet (UV) exposure in this population [16]. All lesions were biopsy-confirmed and treatment-naïve. Exclusion criteria included recent (≤30 days) use of oral or topical antibiotics, systemic immunosuppressants, immunomodulators, topical retinoids, or skin-lightening agents. To minimize external influences on the skin microbiome, participants were instructed to avoid washing or applying cosmetics or skincare products to the sampling area for at least 5 h prior to collection.

Samples were collected immediately prior to the Mohs procedure. For each patient, nine samples were obtained, including one air control and duplicate swabs from four skin sites: the BCC lesion, a perilesional site, a sun-exposed control site at a comparable anatomic location, and a non–sun-exposed control site at a comparable anatomic location. BCC lesions were sampled from the upper back, scalp, postauricular area, nasal ala, and helix. Sun-exposed control samples were collected from the nose, cheek, ear, upper back, and scalp, whereas non-sun-exposed control samples were obtained from the back, chest, and hair-bearing scalp.

Skin swabbing was performed using a standardized protocol to ensure consistent biomass collection across all sampling sites. Sterile FLOQSwabs (Copan Diagnostics, Murrieta, CA, USA) pre-moistened with sterile saline were used to sample a defined 4 cm^2^ area. Each swab was applied with moderate, consistent pressure and rotated for 30 s while moving in a back-and-forth pattern across the sampling area, completing approximately 10 passes. All sample collections were performed by two trained operators (MC and AP) who underwent standardized training to ensure consistency in technique.

The study was conducted in accordance with Good Clinical Practice guidelines and approved by the Henry Ford Health Institutional Review Board.

### 2.2. Whole Genome Shotgun Sequencing

Sample adequacy was assessed prior to library preparation by quantifying extracted DNA using the Qubit dsDNA High Sensitivity Assay Kit (Thermo Fisher Scientific, Waltham, MA, USA). Samples with DNA concentrations below 0.1 ng/μL were flagged as potentially low biomass. DNA input for library preparation ranged from 1 to 10 ng. Whole-genome shotgun sequencing libraries were prepared using the QIAseq FX DNA Library Kit (QIAGEN, Hilden, Germany), with protocol adjustments optimized for low-input DNA. FX Enhancer was included during fragmentation (14 min) to achieve approximately 250 bp fragments. Adapter ligation and PCR amplification conditions were adjusted according to DNA input, with samples 1–3 using 1:1000 adapter dilution and 16 PCR cycles, and samples 4–6 using 1:100 dilution and 14 PCR cycles. Quality control measures included the ZymoBIOMICS Microbial Community Standard (Zymo Research, Irvine, CA, USA) to verify sequencing performance and a no-template control using molecular-grade water to monitor for potential contamination. Sequencing was performed on an Illumina NovaSeq 6000 platform (Illumina, San Diego, CA, USA) using paired-end reads (2 × 151 bp), and reads were demultiplexed using bcl2fastq (v2.17.1.14).

### 2.3. Metagenomic Analysis

Metagenomic preprocessing was performed using KneadData (v0.12.0), incorporating Trimmomatic, Tandem Repeats Finder, Bowtie2, and FastQC [16,17]. Reads were trimmed using sliding window filtering (SLIDINGWINDOW:4:20), repetitive sequences were removed, and human reads were filtered using Bowtie2 with the sensitive-local parameter against the hg37 reference database [17]. Taxonomic classification was performed using Kraken2 [18], with relative abundance estimation using Bracken2 [19]. Functional profiling was conducted using HUMAnN 3.0, with UniRef90 gene families aggregated into MetaCyc pathways [20,21].

### 2.4. Statistical Analysis

All statistical analyses were conducted in R (v4.1). Univariate comparisons were performed using Wilcoxon or Kruskal–Wallis tests as appropriate. Differences in overall microbial community composition were assessed using PERMANOVA via the adonis2 function from the vegan package (v2.6-8) with 10,000 permutations [22]. Linear mixed-effects models were constructed using the lme4 package (v1.1-34) [23], with patient ID included as a random effect and adjustment for age. Differential abundance testing for microbial taxa and MetaCyc pathways was performed using MaAsLin2 (v1.12.0) with Benjamini–Hochberg false discovery rate (FDR) correction [24], using non-lesional samples as the reference group. Given the exploratory nature of the analysis, an FDR threshold of 0.25 was used for discovery, with a tiered interpretive approach that emphasized pathways meeting an FDR < 0.05 and prioritized biologically plausible and consistent findings across sample types.

## 3. Results

A total of four patients with nodular BCC were included, all of whom were White males aged 58–77 years, consistent with the typical demographic distribution of BCC. For each patient, paired sampling was performed across four site types, including BCC, perilesional, sun-exposed non-lesional, and non-sun-exposed non-lesional skin. Following quality control filtering, 2249 microbial species were retained for downstream analysis.

**Figure 1:**
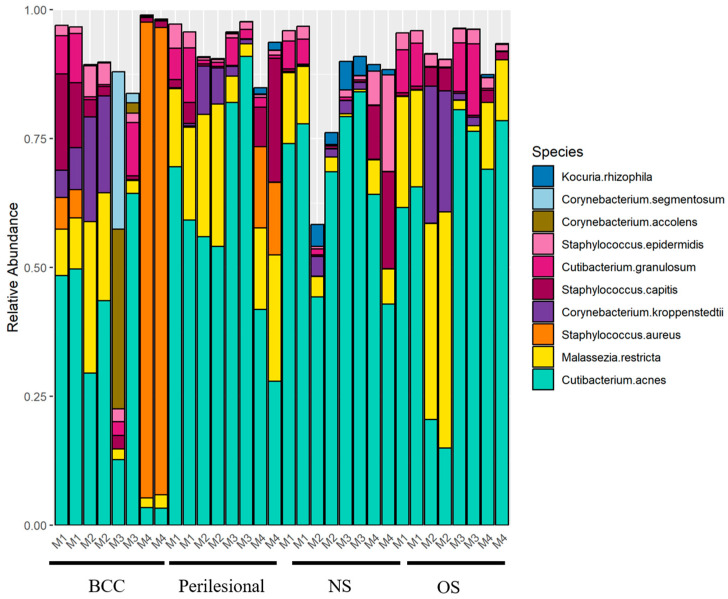
Distribution of the top 10 most abundant microbial taxa across sample types. Relative abundance of the 10 most prevalent taxa is shown for basal cell carcinoma (BCC), perilesional, sun-exposed non-lesional (OS), and non-sun-exposed non-lesional (NS) samples. *Malassezia restricta* is the only fungal species among the top taxa.

The relative abundance of the top 10 microbial taxa across sample types showed that *Cutibacterium acnes* was the most prevalent species across all groups, with a lower relative abundance observed in BCC samples compared to perilesional and control skin (Figure 1). This difference was evaluated using a linear mixed-effects model adjusted for age and accounting for within-patient clustering, which demonstrated a significant reduction in *C. acnes* abundance in BCC samples (β = −0.31, 95% CI: −0.45 to −0.16, *p* = 0.0004). When exponentiated (exp(β) = 0.73, 95% CI: 0.64–0.85), this corresponds to approximately a 27% lower geometric mean relative abundance of *C. acnes* in BCC samples compared to controls. No significant difference was observed between perilesional and control samples (β = −0.02, 95% CI: −0.17 to 0.12, *p* = 0.75).

**Figure 2:**
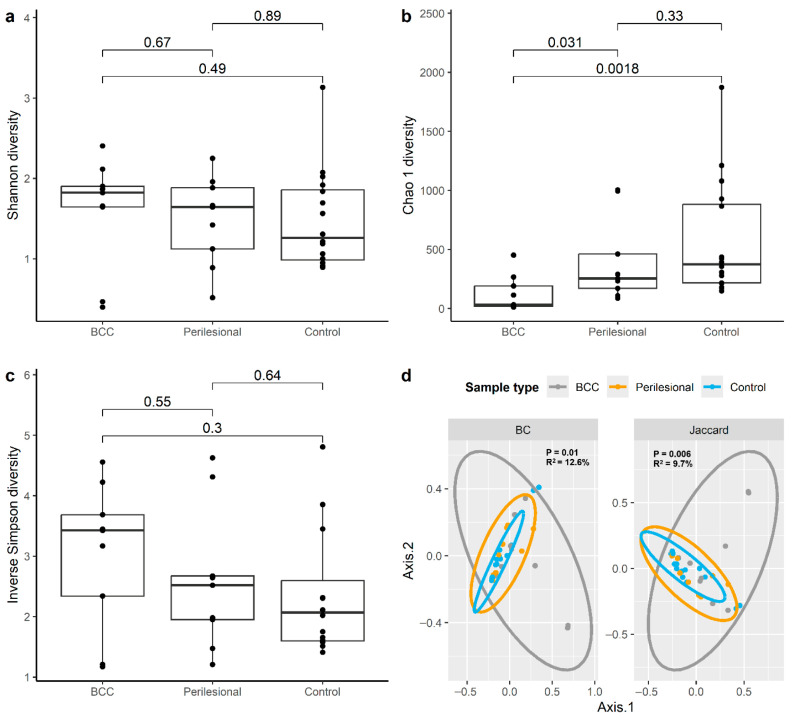
Microbial diversity across sample types. (**a**) Shannon diversity, (**b**) Chao1 richness, and (**c**) inverse Simpson diversity across BCC, perilesional, and control samples. *p* values for alpha diversity comparisons were calculated using the Kruskal–Wallis test. (**d**) Principal coordinate analysis based on Bray–Curtis (BC) and Jaccard distances demonstrating differences in microbial community composition across sample types. Statistical significance for beta diversity was assessed using PERMANOVA.

Assessment of alpha diversity demonstrated that only the Chao1 index differed significantly across sample types (Figure 2a–c; Table 1). Specifically, BCC samples exhibited significantly lower richness compared to controls (estimate = −484.6, 95% CI: −772.1 to −197.2, *p* = 0.003). Perilesional samples showed a non-significant reduction in richness compared to controls (estimate = −242.9, *p* = 0.11). In contrast, Shannon and inverse Simpson indices did not differ significantly across groups (Table 1), consistent with overlapping distributions observed across sample types.

Differences in overall microbial community composition were evaluated using principal coordinate analysis based on Bray–Curtis and Jaccard distances, which demonstrated partial separation of samples by group (Figure 2d). PERMANOVA analysis confirmed that sample type explained a modest but statistically significant proportion of variance in microbial composition (Bray–Curtis R^2^ = 12.6%, *p* = 0.01; Jaccard R^2^ = 9.7%, *p* = 0.01).

Differential abundance analysis at the species level identified four taxa associated with sample status at a false discovery rate threshold of <0.25 (Table 2). These included *Cutibacterium modestum*, *Dermacoccus nishinomiyaensis*, *Cutibacterium acnes*, and *Corynebacterium ihumii*, all of which demonstrated negative coefficients, indicating reduced abundance in BCC samples relative to controls. No taxa were identified as significantly enriched in BCC at this threshold.

**Figure 3:**
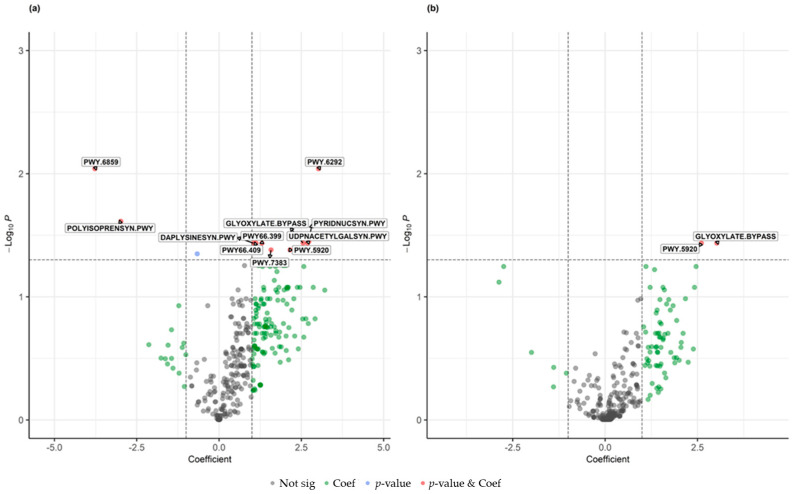
Differential abundance of MetaCyc pathways across sample types. Volcano plots showing pathway-level differential abundance comparing (**a**) control versus BCC samples and (**b**) perilesional versus BCC samples. Each point represents a MetaCyc pathway. Points are colored based on statistical significance and effect size: red, absolute coefficient > 1 and FDR *p*-value < 0.05; blue, FDR *p*-value < 0.05; green, absolute coefficient > 1; grey, not statistically significant.

Functional profiling using the MetaCyc pathway database identified 152 pathways associated with sample status at an FDR threshold of <0.25 (Supplementary Table S1). Comparative analyses of pathway abundance between control and BCC samples and between perilesional and BCC samples demonstrated that a greater number of significant associations were observed in comparisons involving control samples (Figure 3a,b). At a more stringent FDR threshold of <0.05, 11 pathways were significantly associated with BCC relative to control samples, whereas only 2 pathways met this threshold when comparing BCC to perilesional samples, indicating more limited functional differentiation between tumor-adjacent and tumor tissue.

**Figure 4:**
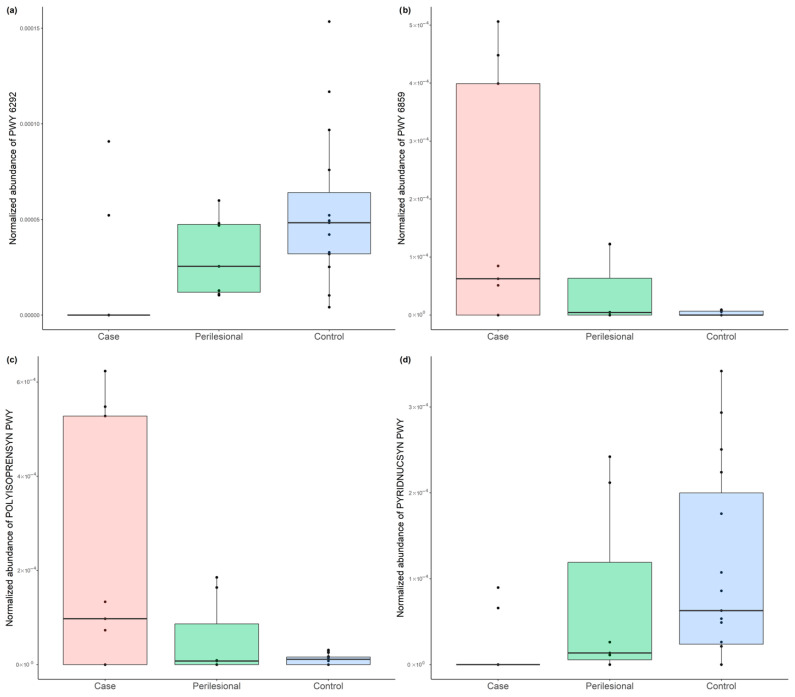
Representative microbial metabolic pathways associated with BCC. Relative abundance of selected MetaCyc pathways across sample types, including (**a**) L-cysteine biosynthesis (mammalian; PWY-6292), (**b**) all-trans-farnesol biosynthesis (PWY-6859), (**c**) polyisoprenoid biosynthesis (*E. coli*; PWY-POLYISOPRENSYN), and (**d**) NAD de novo biosynthesis I (from aspartate; PWY-PYRIDNUCSYN).

To further characterize these pathway-level differences, the most significant and consistently altered pathways across comparisons were examined in greater detail (Figure 4). BCC samples demonstrated decreased abundance of pathways related to L-cysteine and NAD de novo biosynthesis, and increased abundance of pathways related to all-trans-farnesol and polyisoprenoid biosynthesis, relative to both control and perilesional samples. These directional changes were consistent across comparisons involving BCC samples, highlighting reproducible shifts in microbial functional potential at tumor sites.

Collectively, these findings indicate that microbiome alterations in BCC are localized and more pronounced relative to control skin than perilesional tissue.

## 4. Discussion

In this case series of patients with nodular basal cell carcinoma, we identified a localized shift in the cutaneous microbiome between BCC lesions and matched non-lesional skin. BCC samples demonstrated reduced microbial richness, modest but statistically significant shifts in community composition, and depletion of several commensal taxa, most notably *Cutibacterium acnes*. These findings support the presence of a localized tumor-associated microbiome signature, although interpretation is limited by the small sample size.

The reduction in *C. acnes* abundance represents the most prominent taxonomic finding. *C. acnes* is a dominant commensal organism in sebaceous skin and contributes to cutaneous homeostasis through lipid metabolism, maintenance of acidic pH, and modulation of local immune responses [10]. Prior studies in actinic keratosis and cSCC have demonstrated reduced abundance of *C. acnes* in lesional compared to non-lesional skin [6], suggesting that depletion of this organism may be a broader feature of keratinocyte carcinogenesis. Emerging data in BCC also suggest similar alterations in the cutaneous microbiome, including relative reductions in commensal taxa such as *C. acnes*, although these studies are primarily descriptive in nature [25]. However, *C. acnes* abundance is strongly influenced by sebaceous gland density and local microenvironmental conditions, and residual confounding related to anatomic site cannot be fully excluded despite intra-patient matching. The absence of significant differences between perilesional and control samples supports that these changes are localized to tumor sites rather than reflecting a broader field effect.

One potential explanation relates to the role of *C. acnes* in maintaining cutaneous redox balance. *C. acnes* produces the antioxidant protein RoxP, which has been shown to protect against oxidative stress in the skin [11,12]. Given the central role of ultraviolet radiation and reactive oxygen species in BCC pathogenesis, reduced abundance of *C. acnes* may reflect loss of a commensal population or adaptation to tumor-associated microenvironmental changes that contributes to local oxidative homeostasis. Alternatively, these findings may reflect adaptation of the microbiome to tumor-associated alterations in the local microenvironment, including alterations in sebaceous gland function, oxygen tension, and lipid composition [2]. The cross-sectional design of this study limits the ability to determine whether these microbiome changes contribute to tumor development or instead reflect tumor-associated alterations in the local microenvironment.

In addition to taxonomic differences, we observed alterations in microbial functional potential associated with BCC. Specifically, pathways related to L-cysteine biosynthesis and NAD de novo biosynthesis were reduced, whereas pathways related to all-trans-farnesol and polyisoprenoid biosynthesis were increased in BCC samples. These findings suggest shifts in microbial metabolic potential related to redox and lipid-associated processes. These analyses were conducted using an exploratory statistical threshold (FDR < 0.25), with emphasis placed on pathways meeting more stringent criteria, and are based on inferred functional annotation rather than direct measurement of pathway activity or metabolite levels. Accordingly, these findings should be considered hypothesis-generating.

Notably, perilesional samples more closely resembled control skin in diversity analyses, whereas functional profiling suggested comparatively greater similarity between perilesional and BCC samples than between control and BCC samples. This pattern may indicate that broad taxonomic structure is relatively preserved in tumor-adjacent skin, while inferred functional potential shows partial convergence with the tumor-associated microenvironment.

This study has several limitations. The sample size is small and limited to older White males, which may affect generalizability. Although the intra-patient sampling design reduces inter-individual variability, residual confounding related to anatomic site and sebaceous gland density remains possible. In addition, skin swab samples represent low-biomass specimens that are susceptible to variability in sampling yield and possible background contamination, and the high proportion of human DNA in BCC samples further reduced microbial sequencing depth after quality filtering. Finally, the cross-sectional design precludes assessment of temporal relationships or causality.

## 5. Conclusions

This case series demonstrates that basal cell carcinoma lesions are associated with localized reductions in microbial richness, shifts in community composition, and depletion of commensal taxa, including Cutibacterium acnes, compared with matched non-lesional skin. Functional profiling further suggests differences in microbial metabolic potential related to redox- and lipid-associated pathways; however, these findings are based on inferred pathway analyses and should be interpreted with caution. Collectively, these results support the presence of a localized tumor-associated microbiome signature in BCC. Further studies in larger, more diverse cohorts, including longitudinal and mechanistic investigations, are needed to determine whether these microbiome alterations contribute to tumor development or reflect adaptation to the tumor microenvironment.

## Figures and Tables

**Table 1:** Association between alpha diversity indices and sample types.

Alpha Diversity	Sample Type	Estimate	95% CI	*p* Value
Shannon	Control	ref	-	-
	Perilesional	−0.06	−0.50, 0.39	0.81
	BCC	0.004	−0.44, 0.45	0.95
Chao 1	Control	ref	-	-
	Perilesional	−242.9	−530.4, 44.5	0.11
	BCC	−484.6	−772.1, −197.2	**0.003**
Inverse Simpson	Control	ref	-	-
	Perilesional	0.31	−0.58, 1.20	0.51
	BCC	0.64	−0.25, 1.52	0.17

Linear mixed model was used to assess the association between alpha diversity indices and sample types. Patient ID was used as random effect. All models were adjusted for patient age. Bold *p* values indicate significance.

**Table 2:** Differential abundance analysis for sample types.

Species	Value	Coef	SD	*p* Value	FDR *p* Value
*Cutibacterium modestum*	Case	−3.05174	0.690071	0.000154	0.129214
*Dermacoccus nishinomiyaensis*	Case	−3.91958	0.867903	0.000121	0.129214
*Cutibacterium acnes*	Case	−1.5608	0.387891	0.000439	0.183868
*Corynebacterium ihumii*	Case	−2.4446	0.599212	0.00038	0.183868

Patient ID was used as random effect. All models were adjusted for patient age. Control group was the reference in the differential abundance analysis. FDR *p* value < 0.25.

## Data Availability

The original data presented in the study are openly available in the NCBI Sequence Read Archive (SRA) under BioProject ID PRJNA1317643. https://www.ncbi.nlm.nih.gov/bioproject/?term=(PRJNA1317643)%20AND%20bioproject_sra[filter]%20NOT%20bioproject_gap[filter], accessed on 4 September 2025.

## Supplementary Materials

The following supporting information can be downloaded at: https://www.mdpi.com/article/10.3390/microorganisms14040822/s1, Table S1: MetaCyc pathways significantly associated with sample status (FDR < 0.25).

## Abbreviations

The following abbreviations are used in this manuscript:

BCC	Basal cell carcinoma
ROS	Reactive oxygen species
cSCC	Cutaneous squamous cell carcinoma
*C. acnes*	*Cutibacterium**acnes*
